# Positive interactions may decrease cooperation in social dilemma experiments

**DOI:** 10.1038/s41598-018-37674-5

**Published:** 2019-01-31

**Authors:** Lucas Wardil, Ivair R. Silva, Jafferson K. L. da Silva

**Affiliations:** 1 Universidade Federal de Minas Gerais, Departamento de Física, Belo Horizonte, CEP 31270-901 Brazil; 2 Universidade Federal de Ouro Preto, Departamento de Estatística, Ouro Preto, CEP 35400-000 Brazil

## Abstract

A social dilemma appears in the public goods problem, where the individual has to decide whether to contribute to a common resource. The total contributions to the common pool are increased by a synergy factor and evenly split among the members. The ideal outcome occurs if everyone contributes the maximum amount. However, regardless of what the others do, each individual is better off by contributing nothing. Yet, cooperation is largely observed in human society. Many mechanisms have been shown to promote cooperation in humans, alleviating, or even resolving, the social dilemma. One class of mechanisms that is under-explored is the spillover of experiences obtained from different environments. There is some evidence that positive experiences promote cooperative behaviour. Here, we address the question of how experiencing positive cooperative interactions – obtained in an environment where cooperation yields high returns – affects the level of cooperation in social dilemma interactions. In a laboratory experiment, participants played repeated public goods games (PGGs) with rounds alternating between positive interactions and social dilemma interactions. We show that, instead of promoting pro-social behaviour, the presence of positive interactions lowered the level of cooperation in the social dilemma interactions. Our analysis suggests that the high return obtained in the positive interactions sets a reference point that accentuates participants’ perceptions that contributing in social dilemma interactions is a bad investment.

## Introduction

Cooperation is a fundamental ingredient of modern society^1–3^. Cooperation generates benefits at the group level and costs at the individual level^4^. If there are cooperators, it may pay to free-ride on the willingness of others. However, if everyone decides not to cooperate, the outcome is worse than if everyone had cooperated. Under the presumption of instrumental rationality^5^, the solution to the problem is to not cooperate. The public goods game (PGG) reproduces the essential structure of the cooperation problem^6^. Individuals in a group are given an initial endowment *w*. Each one must decide how much of his initial endowment he wants to allocate to public goods production. The sum of contributions is multiplied by *r* (*r* > 1) and distributed evenly to the *n* members of the group. Thus, each one unit contributed to the public goods returns to the individual as *r*/*n*. If *r* < *n*, allocation to the public goods is not beneficial, and the Nash equilibrium solution is to contribute nothing, with final payoffs equal to the initial endowment *w*. However, if everyone had contributed *w*, the outcome would be *rw*, which is larger than the no cooperation solution. On the other hand, if *r* > *n*, each contributed unit returns at a greater value and the Nash equilibrium is to contribute all the initial endowment. In this case, there is no social dilemma and cooperation is the best option both for the individual and the group.

Many features of the environment are likely to have significant effects on the cooperative behaviour of humans, deviating experimental observations from theoretical expectations based on Nash equilibrium analysis^6^. The environment can change the performance criteria, changing the outcome of a cooperative situation. For example, if repetitive interactions are present, reciprocity may arise and the best action today may be cooperation, so tomorrow, the benefit is returned^7^. The environment is represented in an experiment as the exogenous variables that the experimentalist manipulates^6^. The simplest PGG design is the *voluntary contribution mechanism*, where individuals interact in groups and are told to contribute privately from an initial private endowment without any communication with others or any information about what the others are doing. Since the classical PGG experiment done by Isaac, Walker, and Thomas^8^, many environmental factors have been studied within the *voluntary contribution mechanism*. The first variables that were investigated were the multiplicative factor *r* (the multiplicative factor is related to the marginal per capita return), the group size and the number of rounds^6^. A lower multiplicative factor, which is related to the marginal per capita return, yields less cooperation. Group size had no effect in the first experiments, which might be due to the difficulties in disentangling the effects of size and marginal per capita return. In multiple round games, cooperation decreases as more rounds are successively played but hardly reaches the Nash equilibrium of no contribution, showing that humans are more cooperative than predicted by the Nash equilibrium analysis for *r* < *n*. Interestingly, experiments show that humans do not adopt the Nash equilibrium solution even when *r* > *n*. Recall that if *r* > *n*, contributing all the endowment is the best option for both the individual and the group. However, since those that contribute less always outperform those that contribute more, it has been suggested that individuals may exhibit spiteful behaviour^9^.

Most of the first PGG experiments focused on homogeneous environments: all participants were subjected to the same parameters in all rounds. However, heterogeneity also has a significant effect on the evolution of cooperation^10^. One way to implement heterogeneity is to subject participants in the same group to different exogenous variables. Heterogeneity in the endowments and the multiplicative factor were the first types studied. In one experiment, contributions were lower when participants received different endowments than in the symmetric case of equal endowments^11^. In another experiment, some individuals in a group faced a high multiplicative factor, while others faced a low multiplicative factor. Group level contributions resemble an average of what would occur in groups where everyone is exposed to the same multiplicative factor. However, individuals with high and low multiplicative factors contribute differently according to sub-group statistics^12^. Heterogeneity can also be implemented through a variation in the exogenous variables during the rounds. In one experiment, different rates of return from private consumption were randomly assigned to the participants in every round. The parameters were such that the participants could be exposed to interactions where full contribution was the rational decision. The authors found significant evidence for the violation of dominant strategies and warm-growth effect^13^. Although this last experiment exposed participants to games where cooperation is the rational solution, the design was too complex to analyze the interaction between games with different rational solutions.

In the voluntary contribution mechanism, there is no communication among members of the group, making it hard to coordinate the provision of public goods. Even in the absence of coordination, it has been shown that some individuals exhibit conditional cooperation^14^ –conditional cooperators contribute more to a public good the more others contribute. However, due to strategic uncertainty^15^, cooperation may not appear as much as it could because free-riders may undermine the willingness of the conditional cooperators. The presence of conditional cooperators works like a reservoir of potential cooperative behavior: if cooperation takes off, conditional cooperators can boost cooperation. This result was extensively analyzed in the context of evolutionary game theory. Reactive strategies, like the Axelrod tournament winner tit-for-tat^16^, can spread in a population if the number of reciprocators is large enough^17^. The point is that even though we may observe low cooperation at the behavior level, there may be individuals in the group that would be prone to cooperate if the behavior of others were different. Therefore, the way individuals process information about the group is crucial for the decision between cooperation or defection.

The individual’s internal state affects the decision-making process. Many studies have shown that positive emotions, like joy or gratitude, facilitate creativity, cognitive flexibility, innovative responding, and openness to information^18,19^. In particular, experiencing mild positive affection may promote pro-social behaviours like donating to a charity or helping someone pick up dropped papers^20^. Such positive affections can be induced by outside environments. For example, the promotion of pro-environmental behaviour in one environment may, sometimes, increase the likelihood of performing additional pro-environmental behaviours in other environments^21^. Studies on work to family spillover show how well-being is interconnected with environments^22^. While negative emotions, like fear or anger, prompt an individual to have a fast and adapted reaction, positive emotions are thought to broaden perception, thoughts and actions, allowing individuals to develop enduring personal resources^19^. Therefore, we may ask whether a positive cooperative experience can help an individual overcome social dilemmas and sustain cooperation in environments where it is not favoured. By positive, we mean an experience where cooperation yields strong satisfaction.

Here, we investigate how cooperation in social dilemmas is affected by the experience of positive interactions in environments where cooperation is the most rewarding action. Participants played a multiple- round PGG where the multiplicative factor alternated between *r* > *n* (cooperation is the best both for the individual and the group) and *r* < *n* (cooperation is hard to achieve). More specifically, we used *r* < *n* in the odd rounds and *r* > *n* in the even rounds. In the control treatment, we used *r* < *n* in all rounds (see the Methods section for more details). Does the exposure to an environment where investment in the group is strongly incentivized help the solution of the social dilemma? If the satisfaction obtained in the rounds with *r* > *n* spills over to the rounds with *r* < *n*, then we should find that contribution is higher in the heterogenous treatment than in the control treatment. Surprisingly, we find the opposite result: cooperation in the rounds with *r* < *n* is *lower* in the heterogeneous control environment than that in the homogeneous control environment. Experiencing supportive interactions in one environment may decrease cooperation even further in the least supportive environment.

Our results not only push the boundaries of our knowledge of human cooperation a bit further but also help to address the lack of experimental results in non “WEIRD” (Western, Educated, Industrialized, Rich, and Democratic) countries^23^. The control treatment - a simple multiple round PGG - is valuable since it provides support to generalizing conclusions about the evolution of human cooperation. In the Results section, we present our findings, briefly commenting on them. In the Discussion section, we provide explanations regarding our unexpected findings and we establish connections with related research. The details of the experimental design and statistical analysis are provided in the Methods section.

## Results

**Figure 1:**
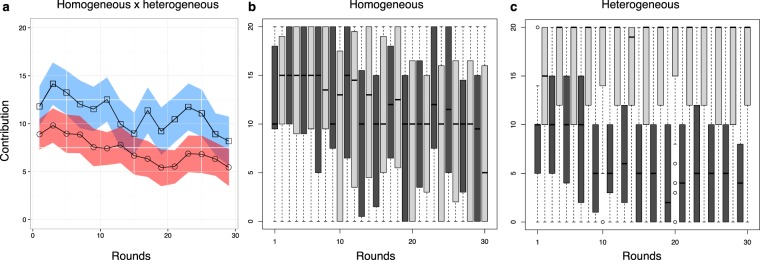
Contributions in all rounds for both treatments. (**a**) Average contribution per round in the homogeneous treatment (☐, blue) and in the heterogeneous treatment (○, red) for *r* = 2. The average contribution in social dilemma games is lower in the heterogeneous treatment than in the homogeneous one. The shaded area defines the 95% confidence interval about the mean. To focus on rounds with *r* = 2, only data for the odd rounds is shown. The (**b**) box plot for the contribution in the homogeneous and **c** in the heterogeneous treatment. Odd rounds are shown in dark grey and even ones in light grey in the box plots.

Figure 1a shows the average contribution per round for both treatments. Although cooperation in the rounds with *r* = 2 in both treatments is higher than zero, as it is usually observed in public goods experiments, the contribution is lower when the participants are exposed alternately to rounds with *r* = 6 than when they are exposed only to rounds with *r* = 2. As expected, cooperation in rounds with *r* = 6 is higher than that in rounds with *r* = 2 because full cooperation yields the maximum return if *r* = 6. Thus, if we look only at the rounds with *r* = 2 in both treatments, we see that cooperation is lower in the heterogeneous environments than in the homogeneous ones.

**Figure 2:**
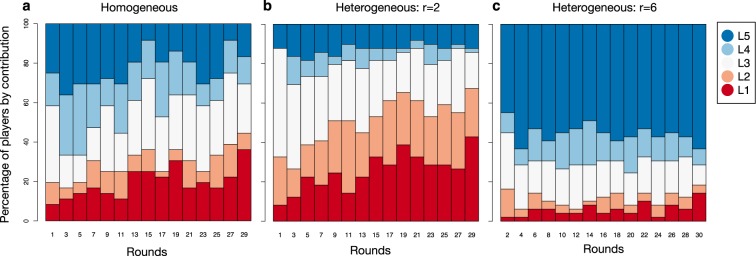
Percentage of players by contribution. The percentage of players in each class of contribution (*L*_1_, *L*_2_, *L*_3_, *L*_4_, and *L*_5_, as defined in the main text) (**a**) in the homogeneous treatment at even rounds, (**b**) in the heterogeneous treatment with *r* = 2 and (**b**) in the heterogeneous treatment with *r* = 6. There are more players adopting the Nash solution of no contribution for *r* = 2 in the heterogenous treatment than in the homogeneous one for most rounds. In addition, fewer players contribute all the initial endowment if *r* = 2 in the heterogeneous treatment. Notice that for *r* = 6 in the heterogeneous treatment, there are many players deviating from the Nash solution of full contribution.

The averaged decline of cooperation conceals a large diversity of behaviours. To simplify the analysis, we classified individuals into five levels of contribution, where *c* is the amount contributed in a round: *L*_1_ = {*c* | *c* = 0}, *L*_2_ = {*c* | *c* ∈ [1, 5]}, *L*_3_ = {*c* | *c* ∈ [6, 14]}, *L*_3_ = {*c* | *c* ∈ [15, 19]}, and *L*_5_ = {*c* | *c* = 20}. Notice that the levels *L*_1_ and *L*_5_ correspond to the Nash equilibrium solutions for *r* = 2 and *r* = 6, respectively. Figure 2 shows the percentage of players by contribution in each round. In the homogeneous treatment, the percentage of players contributing 0 tokens (*L*_1_ level) increases and the percentage contributing 20 tokens (*L*_5_ level) decreases. The percentage contributing intermediate values endures through all rounds. The increase in the percentage of players contributing 0 tokens in the heterogeneous treatment with *r* = 2 is stronger than that in the homogeneous treatment. In addition, the percentage of players contributing 20 tokens in the heterogeneous treatment with *r* = 2 is smaller and shows less temporal variation. The exposure to rounds with *r* = 6 seems to show that contributing 20 is not beneficial in terms of immediate monetary gain and that contributing less is better. In the heterogeneous treatment with *r* = 6, where full cooperation always yields the largest return by definition, we still observe a large percentage of players contributing less than 20 if *r* = 6, as observed in the other experiments^9^.

**Figure 3:**
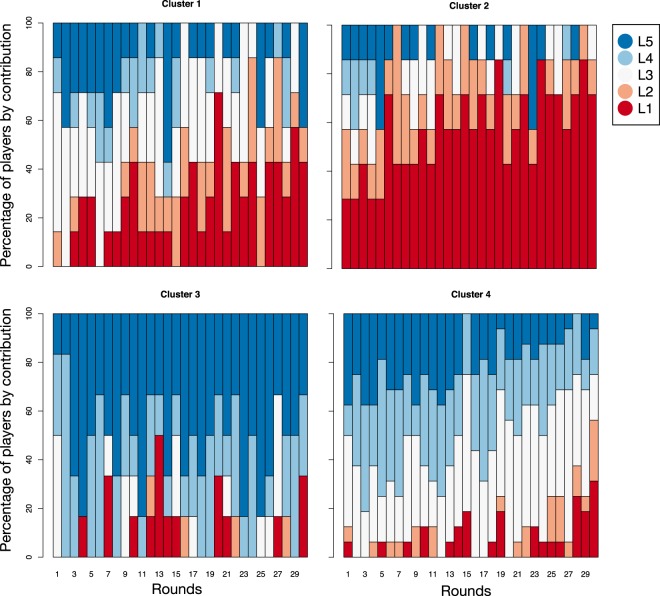
Percentage of players by contribution level in each cluster in the homogeneous treatments. The participants are clustered according to the similarity of their sequence of contribution in the rounds (see the Methods section). There are four clusters of sizes 7, 7, 6 and 16. The only treatment exhibiting significant clustering is the homogeneous treatment, indicating that individual behaviour in the heterogeneous treatment is more diverse. The levels *L*_1_, *L*_2_, *L*_3_, *L*_4_, and *L*_5_ are defined in the main text.

This diversity of contribution raises the question of whether player are conservative or ever-changing strategists. We check whether there are clusters of individuals exhibiting similar behaviour in terms of the contribution per round. We carried out a “conglomerate analysis”, where two individuals are similar if the sequence of their contributions along the rounds is similar (see Methods). Clusters of individuals exhibiting similar behaviour in the homogeneous treatment are shown in Fig. 3, where four clusters were found. There is one cluster (cluster 2) where players are less cooperative in all rounds and two clusters (cluster 3 and 4) where players are more cooperative in all rounds. The remaining cluster (cluster 1) exhibits a large diversity of behaviour. The diversity of behaviour is even larger in the heterogeneous treatment with *r* = 2, where no significant cluster is observed. As expected, the diversity in rounds with *r* = 6 is lower: 61% of the players are in the same cluster of high contributors.

The analysis of self-correlation shows that the contribution in round *n* is significantly correlated the most with round *n* − 1. Therefore, it is reasonable to model the evolution of contributions as a non-homogenous Markov process. The states of the Markov process can, again, be simplified to *E* = {*L*_1_, *L*_2_, *L*_3_, *L*_4_, *L*_5_}. The stochastic variable *X*_*t*_ stands for the contribution of a player in round *t*. There is one transition matrix for each covariate defined by the model. Examples of meaningful covariates are the rounds and the treatment. The transition matrix is then estimated as a best-fit to the experimental data (see the Methods section). First, we check whether the treatment had a significant effect on the evolution of cooperation in the rounds with *r* = 2. We estimate a 5 × 5 transition matrix for each round aggregating both treatments in a single data pool, considering only the odd rounds in the heterogeneous treatment. Then, we considered a second model, where a dummy covariate is added to distinguish the treatments. The likelihood ratio test shows that the treatment had a significant effect on the evolution of cooperation (*P*-value < 10^−6^). That is, the exposure to rounds with *r* = 6 had a significant effect on the decision regarding contribution in rounds with *r* = 2.

**Figure 4:**
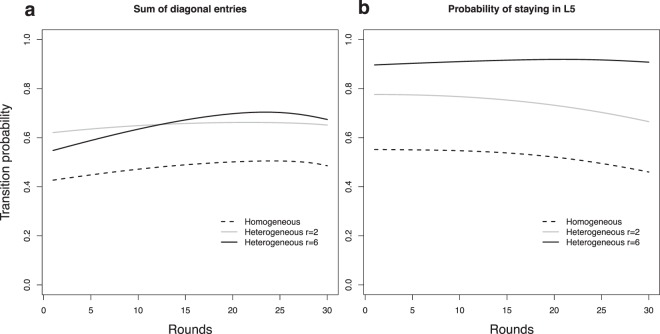
Time evolution of estimated transition probabilities. The sum of the probabilities of staying in the same state is shown in (**a**). In general, individuals in the heterogeneous treatment are more conservative. The time evolution of the probability of continuing to contribute all tokens is shown in (**b**). Notice that, for *r* = 2 in the heterogeneous treatment, high contributors tend to keep contributing all tokens.

The estimated transition matrix for each round provides information on how players change their strategies between rounds. In our model, the Markov process is non-homogeneous, and one matrix is estimated for each round. The transition matrix for round 10 for the homogeneous treatment is shown in Table 1, and the matrix for the heterogeneous treatment with *r* = 2 is shown in Table 2. We see a smaller probability of departure from the high contribution state (*L*_5_) in the heterogeneous treatment with *r* = 2, compared to that of the homogeneous treatment, which corroborates the seemingly constant percentage of high contributors in the heterogeneous treatment with *r* = 2, as shown in Fig. 2. These individuals have a higher probability, 76%, to stay as high contributors in the heterogenous treatment with *r* = 2 than they do in the homogeneous treatment, 54%. The sum of the diagonal elements indicates that individuals in the heterogeneous treatment with *r* = 2 are more conservative, keeping the same level of contribution between rounds. Note that although this conservative behaviour indicates that the same individual is less likely to change his behaviour across rounds, there is considerable diversity among the players, as pointed out in the cluster analysis. These features of the estimated transition probabilities are observed in all rounds, as shown in Fig. 4.

**Table 1:** Estimated transition matrix at round 10 in the homogeneous treatment.

	L1	L2	L3	L4	L5
L1	0.60	0.09	0.16	0.05	0.10
L2	0.14	0.33	0.29	0.09	0.15
L3	0.1	0.10	0.50	0.21	0.08
L4	0.07	0.03	0.23	0.49	0.18
L5	0.16	0.05	0.09	0.16	0.54

The states are the five levels of contribution: *L*_1_, *L*_2_, *L*_3_, *L*_4_, and *L*_5_ (see the main text).

**Table 2:** Estimated transition matrix at round 10 in the heterogeneous treatment, considering only rounds with *r* = 2.

	L1	L2	L3	L4	L5
L1	0.70	0.17	0.11	0.01	0.01
L2	0.15	0.69	0.12	0.03	0.01
L3	0.10	0.12	0.68	0.05	0.05
L4	0.11	0.04	0.27	0.48	0.10
L5	0.10	0.02	0.07	0.05	0.76

Although only the odd rounds are characterized by *r* = 2, the statistical model extrapolates the data so that a comparison between the treatments can be done consistently.

**Figure 5:**
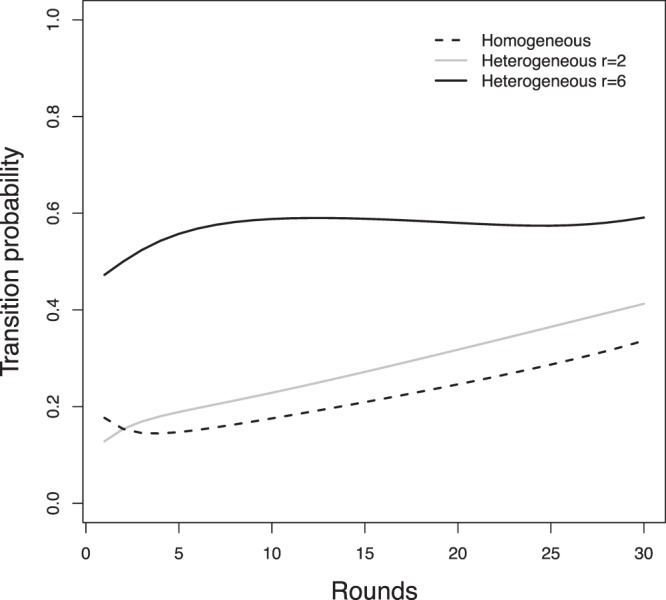
Probability flux towards the Nash equilibrium state. The flux of probability towards the Nash equilibrium state *L*_0_ in rounds with *r* = 2 in both treatments increases as more rounds are played. The flux towards the Nash equilibrium state *L*_5_ in rounds with *r* = 6 in the heterogeneous treatment changes less.

Participants may learn to rationalize the game as more rounds are played. One would expect that as more rounds are played, the flux of probability towards Nash equilibrium states should increase (see the Methods section for the definition of flux). The estimated flux can be calculated by the iteration of the estimated Markov process, taking the first round of the experiment as the initial condition. Figure 5 shows the time dependence of the estimate flux. The flux toward the Nash equilibrium states indeed increases in both treatments for games with *r* = 2.

## Discussion

Our experiment investigates whether there is positive spillover to interactions where cooperation is hard to achieve. Initially, one could expect that a rewarding experience of public goods generation could alleviate the social dilemma of cooperation. One could always have the hope that if individuals are reminded of the social optimum solution, cooperation could be boosted. However, that was not observed in our experiment. Instead, cooperation for *r* = 2 in the heterogeneous treatment was significantly lower than for *r* = 2 in the homogeneous treatment. The participants were asked at the end of the experiment to write down their strategies. It seems that the exposure to games with different multiplicative factors had the effect of classifying rounds with *r* = 2 as bad investments as opposed to rounds with *r* = 6. Therefore, the participants decided to contribute less in rounds with *r* = 2 in the heterogeneous treatments. It appears as if the presence of rounds with *r* = 6 elicits the rationale that typically declines cooperation in PGGs with social dilemma. Data at the individual level indicate that there were diverse behaviours, with some conservative players contributing much or little in all rounds. However, the typical decline in cooperation in multiple-round PGGs for *r* = 2 is clearly observed after averaging the contribution of the participants. This result confirms the clustered behaviour observed in the traditional PGGs, where the participants are typically divided into free-riders, conditional cooperators and other patterns^14^.

The heterogeneity in our experiment differs from the kind of heterogeneity implemented in other experiments, where participants in the same group are subjected to different values of *r*. In these experiments, the contributions resemble an average of what would occur in groups where everyone is exposed to the same multiplicative factor^12^. Interestingly, if the participants in the heterogenous treatment of our experiment had based their rationale on the average value of *r*, which is equal to 4, it would imply a rational decision of full contribution every round–the player does not lose anything by investing all the tokens and, at the same time, increases the odds of the public goods provision. However, full contribution is not observed in our experiment. In addition, the kind of heterogeneity studied in our experiment is different from the kind of heterogeneity usually studied in evolutionary graph theory^24–26^. In these computational studies, heterogeneity means that individuals have many more contacts than others, which typically leads to increased cooperation.

Experiments where cooperation is influenced by the transference of reputation^27,28^ between rounds or by reward/punishment^29^ in subsequent rounds are different from our experiment. If PGGs and reciprocity games are alternated, reputation can be transferred between games and cooperation is boosted in the PGGs. Rewards, more than punishment, promote cooperation in a repeated PGG if player identity persist from round to round^29^. The positive interactions – to have others cooperating with you or to be rewarded – turn the risky action of contributing into an action that may pay off. In our experiment, in contrast, the positive interactions in rounds with *r* = 6 are not a consequence of the individual’s action in the social dilemma rounds. In other words, the positive experience in rounds with *r* = 6 is neither a reward nor a consequence of a good reputation.

Cooperation can be induced if participants are exposed to cooperation-related words^30^ or religious priming^31^, for example. Priming can be defined as “the procedural feature that some previously activated information impacts the processing of subsequent information”^32^. In our experiment, we could expect that the pro-social experience could impact decision-making regarding the social dilemma in a pro-social way, through non-rational ways of information processing. For instance, participants in rounds with *r* = 6 could remember that cooperation can be rewarding. Our experiment excludes this hypothesis. Instead, the mixture of games highlights how much worse it is to cooperate in social dilemma interactions.

One possible theory that could account for our results is reference dependence theory^33^, which states that the value of an action depends on the gains and losses relative to reference points. Surely, the simple analysis of the return of an investment when *r* = 2 would show that each invested unit returns at a lower value. When asked at the end of the experiment about whether maximizing their gains depended on the action of others, almost none of the participants answered that maximization does not depend on the action of others. Thus, it seems that the participants were not consciously driven by the rationale behind the Nash equilibrium solution. Therefore, as no reference point was provided in the homogeneous treatment, the participants defined their strategy based on a single context. On the other hand, the high gains obtained in the heterogeneous treatment with *r* = 6 created a reference point that evinced the losses of contributing in rounds with *r* = 2. Thus, if the participants were risk averse, the increase in the risk caused by the reference point can be the reason we observe a decrease in cooperation. However, we stress that our experiment was not designed to probe reference dependence theory in full. In fact, this theory came out as a possible explanation a posteriori.

Interestingly, research on the influence of positive emotions on risk taking may provide support to our findings. In one experiment, it was shown that the individuals subjected to positive emotions were less willing to engage in high-risk situations but more willing to engage in low-risk situations^34^. One explanation for the lower engagement in high-risk situations was that the individuals were trying to keep up the ongoing positive affective state, implying that the individuals would be more sensitive to losses. Indeed, cooperation in the presence of social dilemmas has an intrinsic strategic uncertainty, since defection is in the best interest of the individual. If the participants perceived the rounds with *r* = 2 as a high-risk situation, then the positive interactions may have induced positive emotions which, in turn, made the individuals less willing to engage in risky cooperative attempts. However, we did not perform any manipulation check to verify the level of risk that the participants associated with the PGG rounds with *r* = 2, and we cannot assert whether the associated risk was high enough so that positive emotions could inhibit cooperation.

The kind of risk intrinsically associated with public goods is different from environmental risk. In traditional PGGs, there is uncertainty regarding what the others will do, the so called “strategic uncertainty”. Environmental risk is an extra risk that can be present if the return from the public good is probabilistic. Experiments in common pool resource dilemmas^15,35^, in public goods with thresholds^36,37^ and in raditional PGGs^38^ have shown that environmental uncertainty decreases cooperation. However, there are some papers suggesting that the effect of uncertainty in public goods games depends on the parametrization of the uncertainty. Under some parametrizations, uncertainty can increase cooperation^39–41^. Different from these experiments, our heterogeneous treatment does not create any environmental risk, and since no communication is allowed, the level of strategic uncertainty is the same in both treatments.

Our initial research hypothesis, that the positive experience obtained in the rounds with *r* = 6 would spill over to the rounds with *r* = 2 and increase cooperation in these rounds was based on many studies suggesting that positive feelings promote helping and generosity. Our experiment provided no evidence to support this claim in the context of public goods. In fact, we find the opposite result. The explanation of our results may rely on the problem of risk aversion. The reference created by the rounds with *r* = 6 may have increased the risks associated with rounds *r* = 2. Similarly, if the risk associated with the *r* = 2 rounds was high enough, the positive emotions may have altered the risk aversion preferences. Therefore, future works should be directed to measure the level of risk aversion of the participants and how it is affected by the alternation in positive interactions.

Promoting cooperation in situations where free-riding is possible is still challenging; determining how to overcome this would greatly benefit society. Our experiment indicates that experiencing interactions where cooperation is good both for the individual and the group, if used as a benchmark to advocate for the goodness of cooperation, may have the opposite effect of actually showing how inferior it is to contribute to public goods generation.

## Methods

### Experiment setup

The public goods game was played in groups of 4. Each group was the same size. Each member of the group received an endowment of 20 tokens at the beginning of each round and could contribute to the public goods any integer amount between 0 and 20. Let us call *c*_*i*,*t*_ the contribution of player *i* in round *t*. The payoff of player *i* in round *t* is given by

1 $${g}_{i,t}=20-{c}_{i,t}+\frac{r{\sum }_{k=1}^{4}{c}_{k,t}}{4},$$

where the sum is for all individuals in the group. For the sake of clarity, let us define the total contribution to the public goods, *G*, as

2 $$G=\sum _{k=1}^{4}{c}_{k,t}.$$

This experiment consists of asking participants to play the public goods game for 30 rounds. Following the traditional setup^8^, the individuals received 20 tokens at the beginning of every round. Notice that this endowment approach differs from the traditional approach adopted in evolutionary game theory, where the contribution cost is usually a fixed value and the payoff of a cooperator is equal to −*c* + *rG*/*N*^42^. We considered two treatments. In the *homogeneous treatment*, the multiplicative factor was set to *r* = 2 in all rounds. In the *heterogeneous treatment*, the multiplicative factor was set to *r* = 2 in the odd rounds and *r* = 6 in the even rounds.

We recruited 85 students from the Universidade Federal de Minas Gerais in Brazil. The participants were split into 3 sessions under the homogeneous treatment (sizes 13, 16 and 7) and 4 sessions under the heterogeneous treatment (sizes 14, 14, and 7).

The participants interacted with the others in the same session through a graphical interface that was built using oTree^43^. The participants in the same session played 30 rounds of the public goods game in groups of 4 players and were subjected to only one treatment. The groups were randomly shuffled each round to minimize the formation of reputations. If the session size was not a multiple of 4, the group with fewest participants was filled with robots that contributed zero tokens.

The focal player *i* could see the record of his/her contributions (*c*_*i*_), payoffs (*g*_*i*_) and total contributions (*G*) from the first round up to the previous one (see Figures S1, S2, and S3 in the Supplementary Information file). In this way, the participants could analyze the trends and the correlations among the contribution, the payoff and social welfare to better rationalize their strategies. Information about the current group was not provided, so there was no way to access the reputation of the others. The identity of the participants was anonymous in all rounds.

The game’s instructions were provided using neutral language and used a questionnaire to ensure that all the participants comprehended the rules, following the standards of experimental game research methodologies (see more information in the Supplementary Information file). Before the game started, the participants were informed that each point would be converted to real money at the end of the game. The conversion rate was set to guarantee the same maximum payoff in both treatments. The average final payoff was approximately R $18.00 (the Brazilian currency is the Real). It is worth stressing that it is likely that the recruitment process had a small bias toward more cooperative subjects, since under Brazilian ethics research guidelines, it is not allowed to announce that the game is worth real money before the provision of the informed consent. Hence, since individuals decided to participate in the absence of any monetary compensation. Therefore, it is likely that our participants have more pro-social preferences. However, since our conclusions are based on the comparison of two treatments, this bias does not compromise our results.

We must say that our experiment does not fully investigate the interaction between the games with *r* = 2 and those with *r* = 6, since there is no homogeneous treatment with *r* = 6 in all rounds. Although we are aware that such a third treatment would improve the understanding of the interaction between games, the basic reason we did not include it was limited funding and logistics issues. Nevertheless, the unidirectional effect of the positive interaction on the social dilemma interactions was consistently explored. This experiment is, to our knowledge, one of the first public goods games systematically done in Brazil, and several challenges had to be addressed.

### Statistical analysis

#### Cluster analysis

Statistical multivariate cluster analysis deals with the stratification of sample units to classify them in groups that are intra-group, similar with respect to P random measurements of interest, but extra-group heterogeneous in the same measurements^44^. Note that in the present paper, the units are the players and the random measurements for the classification are the contributions in the different rounds; here, that is P = 30. A critical point in cluster analysis is the selection of a distance measure, among many proposals available in the literature, that defines the similarity two-by-two of sample units. A highly successful and broadly used method is based on using simple Euclidian distance through complete linkage^45^; hence, this is the approach used in this paper. Due to the hierarchical structure obtained with this method, one can graph the results using the so-called dendrogram^46^. A dendrogram serves as a tree representing the order at which each unit entered in each group, as well as the distances history among the members of the same group.

#### Multi-state Markov model

A multi-state Markov model describes the evolution of a stochastic process with enumerable state space under a continuous time index, t. The transition from state x to state y is defined in terms of a rate *λ*_*x*,*y*_(*t*). This rate is defined as follows:

3 $${\lambda }_{x,y}(t)=\mathop{\mathrm{lim}}\limits_{\delta \to 0}\frac{{\Pr }[{X}_{t+\delta }=y|{X}_{t}=x]}{\delta },\,x,\,y=\mathrm{1,}\,\mathrm{2,}\,\cdots ,K.$$

The *λ*_*x*,*y*_(*t*) values form a *K* × *K* matrix summing up zero by line,. Then ${\lambda }_{x,x}(t)=-\,{\sum }_{y\ne x}{\lambda }_{x,y}(t)$. As this rate varies with t, the process is non-homogeneous in time. In this paper, we modelled the transition rate of each player from one round to another, using the maximum likelihood methodology^47^. This method enables to use covariates to model *λ*_*x*,*y*_(*t*).

The probability flux, *F*, from the set A into the set B of the state space is defined as

$$F(A\to B)=\sum _{i\in A}\sum _{j\in B}\pi (i)P(i,j)\mathrm{.}$$

The flux is the long-run percentage of times that a transition from A to B takes place.

### Ethics statements

This research was approved by the Ethics Board of the Universidade Federal de Minas Gerais under reference CAE 80173017.2.0000.5149. All participants in the experiment provided informed consent. The methods were carried out in accordance with the relevant guidelines and regulations.

## Supplementary information

Supplementary Info

## Data Availability

The datasets generated and analysed during the current study are available in the Open Science Framework repository, https://osf.io/4t8cs/.
