# Supplementary material for: Positive interactions may decrease cooperation in social dilemma experiments
Source: Sci Rep. 2019 Jan 31;9:1017. doi: 10.1038/s41598-018-37674-5 (PMC6355953; doi:10.1038/s41598-018-37674-5)
Supplement: Supplementary file 1 — Supplementary Info [file 41598_2018_37674_MOESM1_ESM.pdf]

SUPLEMMENTARY INFORMATION

POSITIVE INTERACTIONS MAY DECREASE COOPERATION IN SOCIAL DILEMMA EXPERIMENTS

LUCAS WARDIL, IVAIR R. SILVA, AND JAFFERSON K. L. DA SILVA

Contents

1. Recruitment 1

2. Tutorial: instructions to participants 4

3. Data file description 14

1. Recruitment

We sent email to advertise the experiment to all first and second year science students at the Universidade Federal de Minas Gerais (UFMG). The translated email is as follows:

> Hi,
>
> would you be interested to participating in an interesting experiment in game theory?
>
> From day X to day Y, the professores Lucas Wardil and Jafferson K. L. da Silva will run a series of behavioral experiments.

The experiment will take place in a room where you will meet the other participants. You will interact through computer interfaces. You only need to show up one time. The experiment will take around 30 min.

There are some available sessions. If you want to participate, we ask you to register in one that best suits for you. The registration is important, because sessions have limited number of seats.

If you are interest in participating, got to the experiment web site and register for one of the available sessions!

www.siteXXXX.com.br

In this web site you will find more information about the experiment. If you decide to participate, just show up to your chosen session. An email will be sent one day before.

See you,

Lucas Wardil & Jafferson K. L. da Silva

Ps.: If you need any special assistance, please contact us at wardil@fisica.ufmg.br.

In the original language:

Olá,

você estaria interessando em participar de um interessante experimento envolvendo teoria de jogos?

Do dia XX at o dia YY, os professores Lucas Wardil and Jafferson K. L. da Silva conduzirão uma série de experimentos comportamentais.

O experimento será conduzido em uma sala onde você se reunirá com outros participantes. Vocês iro interagir entre si através dos computadores disponíveis. Você só precisa comparecer um única vez. O experimento irá durar aproximadamente 30 min.

Estamos oferencendo diversas sessões e, caso queira participar, pedimos que se registre em uma sessão cujo dia/horário for melhor para você. O pré-registro é importante pois cada sessão possui um número fixo de participantes.

Caso se interesse, vá ao site do experimento e registre-se para uma de nossas sessões disponíveis!

www.siteXXXX.com.br

Nesse site você encontrará mais informações sobre o experimento.

Se você decidir participar, basta comparecer ao local e hora escolhido por você. Um email será enviado com um dia de antecedência para lembrá-lo.

Até mais,

Lucas Wardil & Jafferson K. L. da Silva

Ps.: Caso você apresente alguma necessidade especial, favor entrar em contato com os pesquisadores no email wardil@fisica.ufmg.br para podermos avaliar o caso.

2. Tutorial: instructions to participants

The tutorial comprises 14 pages. The backbone is the same for both treatments. The following sections show the literal translation of the instructions provided to the participants, which were originally provided in brazilian portuguese. Only information regarding the treatment to which the participant was exposed was displayed to the participant.

Page 1. Welcome to the experiment!

First, you must provide written informed consent. The document is on the table. Please, read it carefully before continuing. If you decide to continue, sign the term, leave it on the table and click on “Continue”.

Page 2. In this tutorial you will learn the rules of the game and how to use the graphical interface. You can move back and forth in the tutorial as you go through the tutorial.

You cannot communicate with the other participants during the experiment, including tutorial. If you have any question, please raise your hand and we will assist you.

The experiment starts after all participants finish the tutorial.

Page 3. The game instructions are simple. You will receive an amount of money that depends on your choices and on the other’s choices. Thus, read the instructions carefully. Your performance on the experiment will be accounted as points. The total of points that you get at the end of the experiment will be converted into money and payed to you at the end of the experiment. The conversion factor is 1 point = 3 cents.

Page 4.

Homogeneous treatment

The experiment has 30 rounds. In each round you will interact with 3 other individuals (you + 3). The groups will be shuffled at the beginning of each round, that is, in each round you will likely interact with different individuals.

In the beginning of all rounds, you will receive 20 points. The other members of the group will also receive 20 points in the beginning of all rounds. Your task is to decide what you want to do with your points. You have to decide how much you want to invest in a group project and how much you want to keep for yourself.

The amount that you decide to invest in the project will be added to the amount invested by the other members of the group. The total invested will be multiplied by a factor. The final amount, after multiplication, will be evenly split among all in the group.

Example:

If all in the group decide to contribute 20 points, the total invested in the project is 80 points. This quantity is multiplied be 2, resulting in 160. Each will receive an equal amount from the project, that is, each will receive 40 points (160 points divided by 4 players).

Heterogeneous treatment

The experiment has 30 rounds. In each round you will interact with 3 other individuals (you + 3). The groups will be shuffled at the beginning of each round, that is, in each round you will likely interact with different individuals.

In the beginning of all rounds, you will receive 20 points. The other members of the group will also receive 20 points in the beginning of all rounds. Your task is to decide what you want to do with your points. You have to decide how much you want to invest in a group project and how much you want to keep for yourself.

The amount that you decide to invest in the project will be added to the amount invested by the other members of the group. The total invested will be multiplied by a factor. The final amount, after multiplication, will be evenly split among all in the group.

The multiplicative factor changes throughout the game. In the odd rounds, the multiplicative factor is equal to 2. In the even rounds, the multiplicative factor is equal to 6.

Example:

Suppose you are in an even odd. If all in the group decide to contribute 20 points, the total invested in the project is 80 points. This quantity is multiplied be 2, resulting in 160. Each will receive an equal amount from the project, that, each will receive 40 points (160 points divided by 4 players).

Page 5.

Homogeneous treatment

In the next pages you will answer a few simple questions about the game. To continue in the experiment, you must answer the questions correctly. The rules of the game will be available at the top of the page. If you wish, you can use the computer calculator.

Heterogeneous treatment

In the next pages you will answer a few simple questions about the game. To continue in the experiment, you must answer the questions correctly. The rules of the game will be available at the top of the page. If you wish, you can use the computer calculator.

The rules will be the same in all rounds, but the multiplicative factor will change:

- odd rounds: multiplicative factor is equal to 2

- even rounds: multiplicative factor is equal to 6

Page 6.

Homogeneous treatment

Rules of the game

In each round you will interact with 3 other individuals (you + 3). In the beginning of all rounds, you will receive 20 points. The other members of the group will also receive 20 points in the beginning of all rounds. Your task is to decide what you want to do with your points. You have to decide how much you want to invest in a group project and how much you want to keep for yourself.

The amount that you decide to invest in the project will be added to the amount invested by the other members of the group. The total invested will be multiplied by a factor. The final amount, after multiplication, will be evenly split among all in the group.

The total of points of each participant at the end of each round will be equal to the sum of the points that the participant kept for himself plus the amount received from the group project.

Question 1

If all, including you, contribute 10 points, then:

(1) The amount of points that you keep for yourself is equal to .....
(2) The amount of points that you will receive from the project is equal to .....
(3) The total of points at the end of the round will be equal to .....

Heterogeneous treatment

Rules of the game

In each round you will interact with 3 other individuals (you + 3). In the beginning of all rounds, you will receive 20 points. The other members of the group will also receive 20 points in the beginning of all rounds. Your task is to decide what you want to do with your points. You have to decide how much you want to invest in a group project and how much you want to keep for yourself.

The amount that you decide to invest in the project will be added to the amount invested by the other members of the group. The total invested will be multiplied by a factor. The final amount, after multiplication, will be evenly split among all in the group.

The multiplicative factor changes throughout the experiment. In the odd rounds, the multiplicative factor is equal to 2. In the even rounds, the multiplicative factor is equal to 6.

The total of points of each participant at the end of each round will be equal to the sum of the points that the participant kept for himself plus the amount received from the group project.

Question 1A

Suppose you are in an odd round. The multiplicative factor is equal to 2. If all, including you, contribute 10 points, then:

(1) The amount of points that you keep for yourself is equal to .....
(2) The amount of points that you will receive from the project is equal to .....
(3) The total of points at the end of the round will be equal to .....

Question 1B

Suppose you are in an even round. The multiplicative factor is equal to 6. If all, including you, contribute 10 points, then:

(1) The amount of points that you keep for yourself is equal to .....

(2) The amount of points that you will receive from the project is equal to .....

(3) The total of points at the end of the round will be equal to .....

Page 7.

Homogeneous treatment

Rules of the game

Omitted here, in the manuscript, but shown in the experiment. The same as in page 6.

Question 2

If all, including you, contribute 20 points, then:

(1) The amount of points that you keep for yourself is equal to .....

(2) The amount of points that you will receive from the project is equal to .....

(3) The total of points at the end of the round will be equal to .....

Heterogeneous treatment

Rules of the game

Omitted here, in the manuscript, but shown in the experiment. The same as in page 6.

Question 2A

Suppose you are in an odd round. The multiplicative factor is equal to 2. If all, including you, contribute 20 points, then:

(1) The amount of points that you keep for yourself is equal to .....

(2) The amount of points that you will receive from the project is equal to .....

(3) The total of points at the end of the round will be equal to .....

Question 2B

Suppose you are in an even round. The multiplicative factor is equal to 6. If all, including you, contribute 20 points, then:

(1) The amount of points that you keep for yourself is equal to .....
(2) The amount of points that you will receive from the project is equal to .....
(3) The total of points at the end of the round will be equal to .....

Page 8.

Homogeneous treatment

Rules of the game
Omitted here, in the manuscript, but shown in the experiment. The same as in page 6.

Question 3

If all, including you, contribute 0 points, then:

(1) The amount of points that you keep for yourself is equal to .....
(2) The amount of points that you will receive from the project is equal to .....
(3) The total of points at the end of the round will be equal to .....

Heterogeneous treatment

Rules of the game
Omitted here, in the manuscript, but shown in the experiment. The same as in page 6.

Question 3A

Suppose you are in an odd round. The multiplicative factor is equal to 2. If all, including you, contribute 0 points, then:

(1) The amount of points that you keep for yourself is equal to .....

(2) The amount of points that you will receive from the project is equal to .....

(3) The total of points at the end of the round will be equal to .....

Question 3B

Suppose you are in an even round. The multiplicative factor is equal to 6. If all, including you, contribute 0 points, then:

(1) The amount of points that you keep for yourself is equal to .....

(2) The amount of points that you will receive from the project is equal to .....

(3) The total of points at the end of the round will be equal to .....

Page 9.

Homogeneous treatment

Rules of the game
Omitted here, in the manuscript, but shown in the experiment. The same as in page 6.

Question 4

If all the other participants contribute 20 points and you contribute 0 points, then:

(1) The amount of points that you keep for yourself is equal to .....

(2) The amount of points that you will receive from the project is equal to .....

(3) The total of points at the end of the round will be equal to .....

Heterogeneous treatment

Rules of the game

Omitted here, in the manuscript, but shown in the experiment. The same as in page 6.

Question 4A

Suppose you are in an odd round. The multiplicative factor is equal to 2. If all the other participants contribute 20 points and you contribute 0 points, then:

(1) The amount of points that you keep for yourself is equal to .....
(2) The amount of points that you will receive from the project is equal to .....
(3) The total of points at the end of the round will be equal to .....

Question 4B

Suppose you are in an even round. The multiplicative factor is equal to 6. If all the other participants contribute 20 points and you contribute 0 points, then:

(1) The amount of points that you keep for yourself is equal to .....
(2) The amount of points that you will receive from the project is equal to .....
(3) The total of points at the end of the round will be equal to .....

Page 10.

Homogeneous treatment

Rules of the game

Omitted here, in the manuscript, but shown in the experiment. The same as in page 6.

Question 5

If all the other participants contribute 0 points and you contribute 20 points, then:

(1) The amount of points that you keep for yourself is equal to .....
(2) The amount of points that you will receive from the project is equal to .....

(3) The total of points at the end of the round will be equal to .....

Heterogeneous treatment

Rules of the game

Omitted here, in the manuscript, but shown in the experiment. The same as in page 6.

Question 5A

Suppose you are in an odd round. The multiplicative factor is equal to 2. If all the other participants contribute 0 points and you contribute 20 points, then:

(1) The amount of points that you keep for yourself is equal to .....
(2) The amount of points that you will receive from the project is equal to .....
(3) The total of points at the end of the round will be equal to .....

Question 5B

Suppose you are in an even round. The multiplicative factor is equal to 6. If all the other participants contribute 0 points and you contribute 20 points, then:

(1) The amount of points that you keep for yourself is equal to .....
(2) The amount of points that you will receive from the project is equal to .....
(3) The total of points at the end of the round will be equal to .....

Page 11. Now you will learn to interact with the graphical interface.

You will visit two pages per round. In the first page, you will inform the amount that you want to invest in the group project. In the second page, after all in your group informed their investments, you will visualize the total of points that you obtained in the round.

The two pages that you will interact with are shown bellow:

Figure 1: Investment page in the homogeneous treatment

Figure 2: Results page in the homogeneous treatment.

Page 12. The history of you action can be visualized graphically in both pages. The same graphics is shown in both pages.

Figure 3: History in the homogeneous treatment.

Figure 4: History in the heterogeneous treatment.

Page 13. Every time you move to the next round, you will have to wait for the others to finish the current round. While you wait, you will see a waiting page.

Page 14. You have reached the end of the tutorial. Click in the link bellow to start the experiment. You will star the first round immediately.

3. Data file description

The datasets generated and analysed during the current study are available in the Open Science Framework repository `https://osf.io/4t8cs/`. The data is available on csv format. A single table contains information on all session. Each line contains information about the action of one participant in one round, as well as information of what happened in that round. In the following we describe each column.

participant.id_in_session: the participant's id id within the session.

participant.code : the unique participant's ID in the experiment.

subsession.round_number : the round.

group.id_in_subsession : the group ID

group.current_efficiency : the multiplicative factor r.

player.contribution: the contribution c_i of the player.

group.total_contribution : the total of contributions T.

group.individual_share : more specifically, $\frac{r\sum_{k=1}^{4} c_k}{4}$ in the round.

player.payoff: payoff g_i.

player.cumulative_payoff: the cumulative payoff. It is the sum of the payoff from the first to the current rounds.

session.code: the session id

session.treatment: the treatment

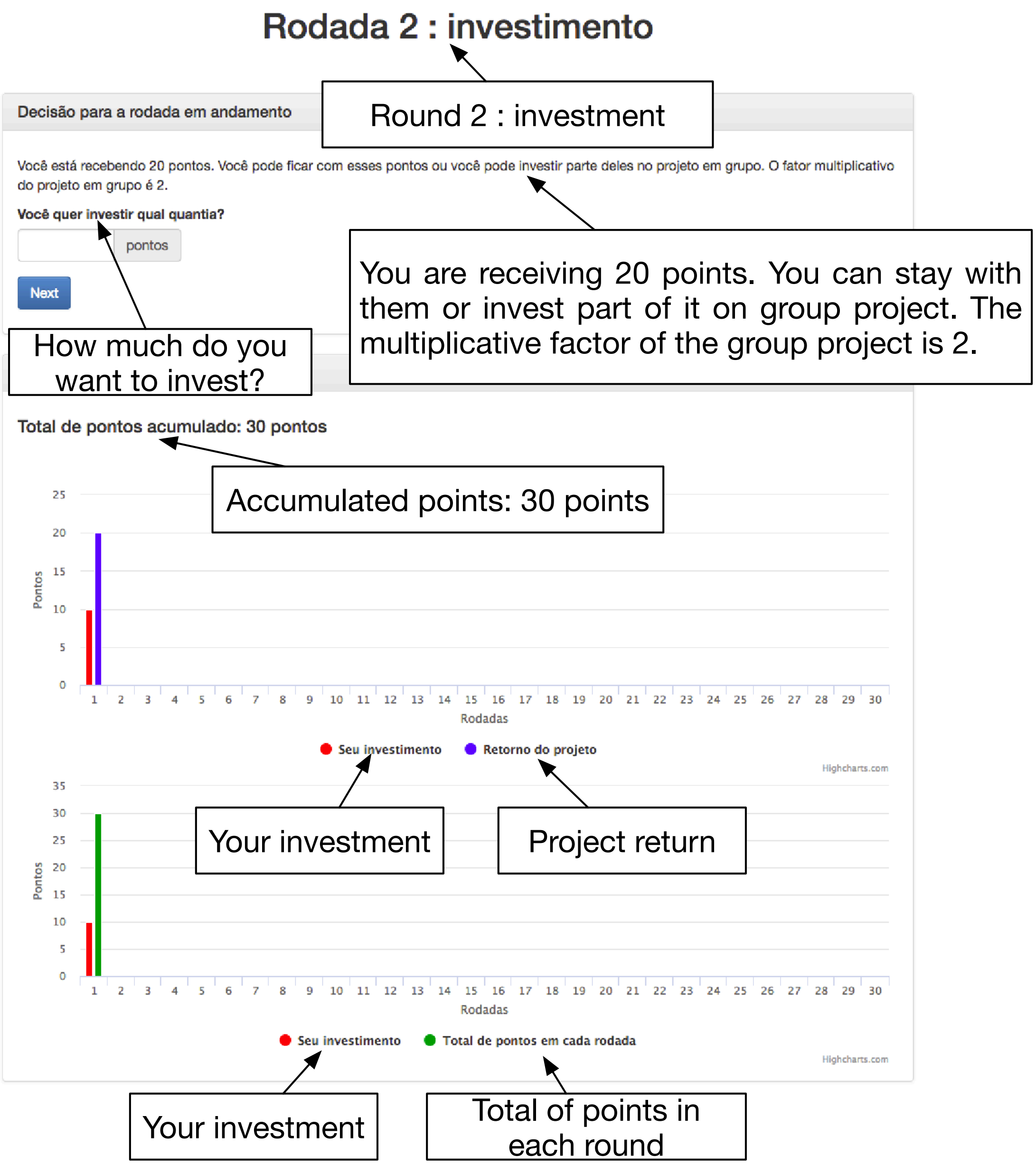

FIGURE S1. Contribution page in the homogeneous treatment.

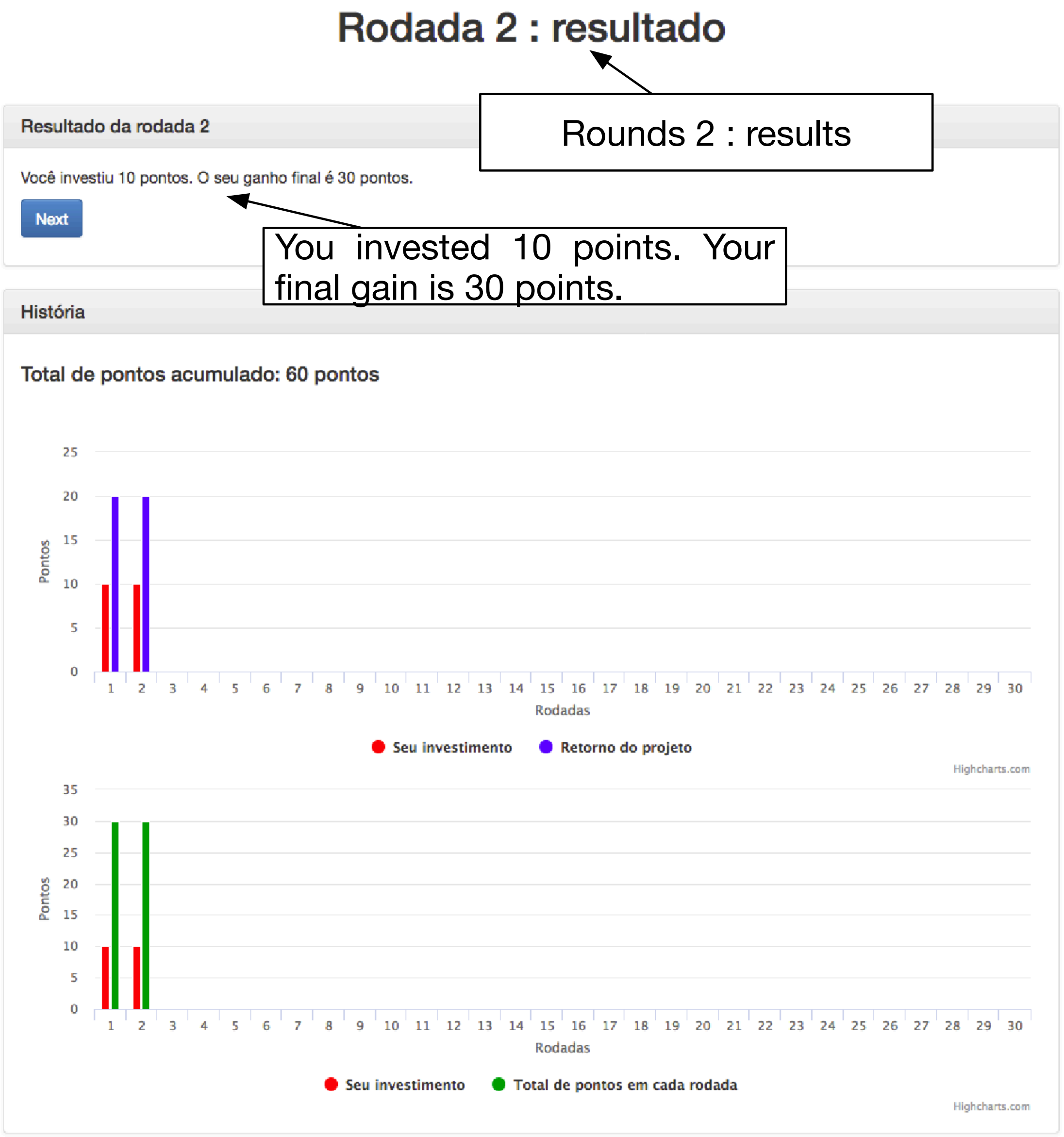

FIGURE S2. Results page in the homogeneous treatment.

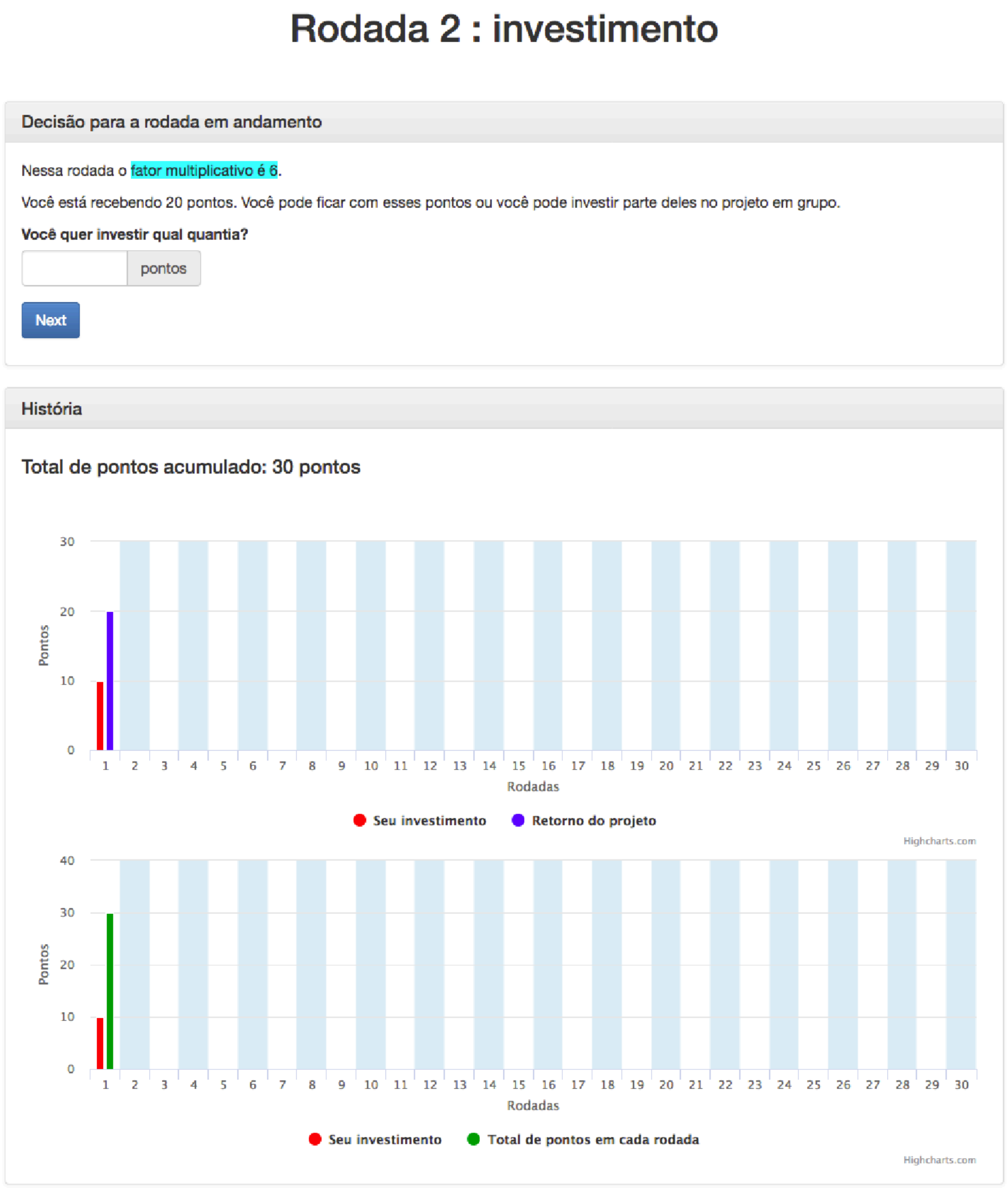

FIGURE S3. Contribution page in the heterogenous treatment.
